# Using ERPs to explore the impact of affective distraction on working memory stages in schizophrenia

**DOI:** 10.3758/s13415-018-0578-4

**Published:** 2018-04-13

**Authors:** Łukasz Okruszek, M. Jarkiewicz, M. Gola, M. Cella, E. Łojek

**Affiliations:** 1 Clinical Neuroscience Lab, Institute of Psychology, Polish Academy of Sciences, Jaracza 1, 00-378 Warsaw, Poland; 2 Third Department of Psychiatry, Institute of Psychiatry and Neurology, Warsaw, Poland; 3 Department of Neurophysiology, Institute of Psychiatry and Neurology, Warsaw, Poland; 4 Centre for Modern Interdisciplinary Technologies, Nicolaus Copernicus University, Torun, Poland; 5 Department of Psychology, Institute of Psychiatry, Psychology & Neuroscience, King’s College London, London, UK; 6 Faculty of Psychology, University of Warsaw, Warsaw, Poland

**Keywords:** Schizophrenia, Working memory, Emotion, ERP

## Abstract

Research on individuals with schizophrenia (SCZ) shows a variety of emotional and cognitive deficits. We examined the hypothesis that ineffective emotional interference control may impact working memory (WM) performance by disrupting information encoding, maintenance, or retrieval in SCZ. Twenty-eight SCZ and 28 matched healthy controls (HC) performed the visual and verbal delayed-matching-to-sample task (DMST) with trials preceded by negative and nonemotional visual distractors. Event-Related Potentials associated with affective stimuli processing (Late Positive Potential-LPP) and WM-encoding (target-P3), maintenance (Negative Slow Wave-NSW), and retrieval (probe-P3) were analyzed. Patients showed overall worse DMST accuracy than HC. Emotional distraction negatively impacted accuracy during the verbal DMST in both groups combined. Both groups also displayed similar LPP modulation during the presentation of emotional distractors. HC showed enhanced NSW after presentation of a negative distraction, whereas this did not occur in SCZ. Comparable effects of emotional distraction were found for WM-encoding and retrieval in both groups. While emotional and neutral stimuli differentially impacted WM-maintenance on the neural level in HC, we did not observe this effect in SCZ, even though both groups showed similar behavioral and neurophysiological reactions to affective stimuli. Deficits in inhibitory mechanisms in SCZ may be responsible for this effect and may have particular relevance for WM-maintenance difficulties.

**Electronic supplementary material:**

The online version of this article (10.3758/s13415-018-0578-4) contains supplementary material, which is available to authorized users.

## Introduction

There is increasing convergence on the view that cognitive and emotional processes are interdependent (Storbeck & Clore, 2008). A number of studies have provided the evidence for the impact of task-irrelevant emotional distraction on concurrent attentional, working memory, and executive tasks (Mueller, 2011; Storbeck & Clore, 2011), as well as in long-term memory and learning (Storbeck & Clore, 2011). At the same time, research in affective neuroscience has led to the reconsideration of the traditional distinction between “emotional” (subcortical) and “cognitive” (cortical) structures of the brain. Because each complex behavior is a reflection of the dynamic interaction between many brain networks, the contemporary view is that none of the brain regions or structures should be deemed as uniquely responsible for emotional or cognitive processes (Pessoa, 2008). This provides an explanation of how emotions may influence cognitive processes. For example, the impact of situational emotional arousal on episodic memory (“flashbulb memories”) is now explained by the modulatory interaction between the amygdala and the hippocampus (Dolcos, Iordan, & Dolcos, 2011). Similarly, the impact of emotional distraction on complex cognitive processes (e.g., working memory) is now attributed to the negative functional connectivity between the frontoparietal network (“Cold Ex”: dorsolateral prefrontal cortex, lateral parietal cortex) and limbic network (“Hot Emo”: amygdala, orbitofrontal and medial prefrontal cortex) (Dolcos et al., 2011; Ray & Zald, 2012).

A wealth of research has shown that patients with schizophrenia (SCZ) experience emotional (Green, Horan, & Lee, 2015) and cognitive (Green et al., 2015; Reichenberg & Harvey, 2007) deficits. However, most of the research in this field has largely considered these phenomena as separate and did not account for the bilateral relationships between both domains (Anticevic & Corlett, 2012). One example of this is research on working memory, which, according to meta-analytic findings, is among the cognitive domains that are most affected in SCZ (Reichenberg & Harvey, 2007). Working memory is a core system for transient storage and processing of any internal or external information. Its effective functioning is necessary for multiple cognitive domains, including learning, reasoning, and language comprehension. Accordingly, it has been previously suggested that working memory deficits may underlie other cognitive dysfunctions (Lawlor-Savage & Goghari, 2014) and negative symptoms observed in SCZ (Gold, Waltz, Prentice, Morris, & Heerey, 2008). Working memory deficits also have been linked to multiple factors associated with poor functional outcomes in patients, including poor community and social functioning, low self-care ability, and problems with returning to work (Rajji, Miranda, & Mulsant, 2014). Additionally, studies have associated working memory neural mechanisms with frontoparietal network activation (Owen, McMillan, Laird, & Bullmore, 2005), thus making it particularly vulnerable for possible emotional interference.

Patients with schizophrenia have an intact ability to react to emotional stimuli “in-the-moment” (Kring & Moran, 2008; Okruszek et al., 2016). This would suggest that a similar impact of emotional stimuli on concurrent cognitive processes should be observed in both patients and in healthy individuals. However, the strongest conclusion from neuroimaging studies on the impact of emotional processing on working memory is that, while in HC prefrontal control mechanisms can be overcome only by highly salient emotional stimuli, SCZ are prone to distraction by both emotional and nonsalient neutral stimuli. Diaz et al. (2011) found larger bilateral posterior cingulate, bilateral orbital frontal cortex, and right parietal cortex response for negative compared with neutral stimuli presented during a verbal memory task in HC but not in SCZ. In another fMRI study based on a visual working memory task, HC exhibited a modulation of interference effects depending on the intensity of the negative distraction, whereas SCZ showed reduced neural activity associated with interference control regardless of the interference intensity (Anticevic, Repovs, Corlett, & Barch, 2011). Additionally, reduced prefrontal activation was observed in SCZ in the context of normal limbic system activation.

The limited temporal resolution of neuroimaging studies prevents us from determining when the effects of the emotional distraction are most accentuated. This is an important issue, because different approaches may be used to strengthen cognitive control in each step of working memory processing. Additionally, emotional stimuli are usually presented during the working memory maintenance phase, thus preventing the examination of the impact of emotion on working memory encoding or retrieval. Previous studies have shown that, depending on the timing of the distraction, different working memory-processes may be impacted (Hahn et al., 2010). Thus, exploring the influence of emotional arousal on each of these working memory components would require presentation of alternating emotional stimuli and a working memory task combined with a high temporal resolution imaging method, such as EEG.

Various versions of the classic Sternberg matching-to-sample task have been used to study the neurophysiology of working memory in HC (Li, Chan, & Luo, 2010) and more recently in SCZ (Zhao et al., 2011). A study comparing SCZ and HC during a short-term memory scanning task revealed between-group differences during multiple working memory stages in SCZ: (i) decreased early visual potentials, (ii) increased P3 at encoding, (iii) negative deflection during some of the subperiods of the maintenance phase, and (iv) increased P3 at retrieval (Zhao et al., 2011). On the other hand, a study that examined the effects of additional task-related distraction presented during the delayed response task revealed that specific enhancement of retention-related slow waves may be observed under additional task demands in HC but not in SCZ (Cameron et al., 2003). To examine the stage effects of negative emotion on working memory, Li and collaborators (Li et al., 2010) recorded EEG while HC performed either a verbal or spatial delayed-matching to sample task (DMST) with distracting affective pictures. The effects of negative emotion were seen in P3 potentials associated with working memory encoding and retrieval while a differential impact of negative versus neutral distraction on spatial and verbal working memory was observed only during the working memory maintenance stage.

These results allow us to hypothesize that the discrepant patterns of impact of emotion on working memory in SCZ and HC observed previously in neuroimaging studies may be seen at multiple stages of working memory processing. Furthermore, various phases of working memory may be differentially affected by the deficient interference control mechanisms in SCZ. We explored this problem by examining the neurophysiological correlates of subsequent working memory stages (encoding, maintenance, retrieval) under the conditions of affective distraction in HC and in SCZ.

## Methods and materials

### Participants

We examined 28 patients with a diagnosis of schizophrenia according to the ICD-10 and meeting the following inclusion criteria: 1) age between 18-50 years; 2) stable medication regimen (at least 2 weeks). Exclusion criteria included: 1) any history of comorbid neurological or psychiatric disorders; 2) diagnosis of intellectual disability; 3) history of drug use within the 3 months before participation in the study; and 4) current use of benzodiazepines, antihistamines, or anticholinergics. The sample of patients consisted of 8 inpatients and 20 outpatients. Patients were assessed with The Positive and Negative Syndrome Scale (PANSS, (Kay, Fiszbein, & Opler, 1987) by a qualified psychiatrist before participation in the study. PANSS scores were calculated according to the five-factor solution of van der Gaag et al. (2006). Only patients with an established diagnosis of schizophrenia according to ICD-10 criteria were recruited in this study. None of the patients included in the study fulfilled the diagnosis of affective or anxiety disorders. The diagnosis was verified by the available documentation and was confirmed by a clinical interview conducted by a qualified psychiatrist (MJ), who also screened participants for exclusion criteria. Twenty-eight control participants matched for age and gender were recruited via an online platform. None of the participants disclosed any neurological or psychiatric disorder. The study procedure was approved by the University of Warsaw Ethics Committee.

### Procedure

#### Affective stimuli

A total of 120 pictures selected from the Nencki Affective Picture System (NAPS; (Marchewka, Zurawski, Jednoróg, & Grabowska, 2014) were used as a distraction during the DMST. NAPS is a set of high-quality affective pictures divided into five thematic categories (i.e., objects, landscapes, animals, people, faces). Each of the pictures has normative valence (negative-positive) and arousal (relaxed-aroused) ratings collected from 204 healthy participants (Marchewka et al., 2014).

According to the normative ratings, half of the pictures selected for the study (N = 60) were classified as negative and highly arousing (NEG), whereas the remaining pictures were rated as neutral (i.e., nonarousing and of neutral valence [NEU]). A recent paper has emphasized the relevance of the content for affective processing in SCZ (Bodapati & Herbener, 2014), so half of the pictures of each type were drawn from categories with social content (people/faces) and the other half from categories without social content (animal/object). No differences between NEG and NEU were observed in regard to their visual characteristics (contrast (NEG: 65.9 ± 10.1 vs. NEU 67.9 ± 10.9; F(1,119) = 1.1; not significant [n.s.]), luminance (NEG: 121.1 ± 34.0 vs. NEU: 120.8 ± 26.0 F(1,119) < 0.01; n.s.) or entropy, which is calculated from the histogram distribution of the 8-bit, gray-level intensity values. This is considered indicative for the “randomness” of the image; higher entropy was associated with higher pixel-to-pixel variability (Marchewka et al., 2014) (NEG: 7.5 ± 0.4 vs. NEU: 7.6 ± 0.3 F(1,119) = 2.6; n.s.). The full list of the stimuli can be seen in Supplementary Table 1.

#### Delayed matching to sample task

The working memory task used in this study is a modification of the delayed-matching-to-sample (DMST) task from (Li et al., 2010), created with STIM2 software (Compumedics, Australia). Each trial started with presentation of an affective picture from the NAPS battery for 1,000 ms and presentation of a fixation cross for the next 400-600 ms.

During the encoding phase, a set of the three capital letters drawn from a subset of 12 letters from the Latin alphabet was simultaneously presented for 300 ms. Each of the letters was allocated to a different location, which were drawn out of the 12 possible on-screen coordinates. Locations were set by the radius of an imaginary circle (either 2 cm or 6 cm), starting in the center of the screen and incrementally moving by 60 degrees from a horizontal position. The participant was instructed to either remember which three letters were presented (VERBAL) or to remember where the three letters were presented (SPATIAL).

After the presentation of the target letters, only the fixation cross remained on-screen for the next 4,000-4,300 ms (maintenance phase). After that period, a single letter (probe) was shown for 300 ms (retrieval phase). During the VERBAL task, participants were instructed to respond by pressing one of the two buttons if the probe letter was among the three letters presented during the encoding phase of the trial. Similarly, during the SPATIAL task participants were instructed to respond if the probe letter was presented in the same location as any of the three letters from the encoding phase. Responses were collected during the probe presentation (300 ms) and the subsequent 1,500-ms period during which the fixation cross remained visible on-screen. If no response was given during that time, the next trial was presented. The schematic structure of the task can be found in the Supplementary Materials (Fig. S1).

Overall, 120 stimuli were used for this study. Each stimulus was presented twice, once with VERBAL instruction and once with SPATIAL instruction, producing 240 trials in total. Additionally, 60 trials of each type were combined with either neutral (NEU) or negative (NEG) distraction pictures, thus producing four different task conditions presented in a block design. In half of the trials from each condition, the probe letter matched one of the target stimuli. Condition order was counterbalanced between subjects. The DMST was presented on a 17’ CRT monitor with participants seated approximately 75 cm from the screen.

Upon completion of the DMST procedure, participants were asked to provide ratings for the affective stimuli. During the rating procedure, which was created with PsychoPy (Peirce, 2007), pictures were presented in a fully randomized order. Each picture was displayed with two rating scales ranging from “relaxed-aroused” and “negative-positive,” respectively. Corresponding Self-Assessment Manikin (SAM; (Bradley & Lang, 1994)), illustrations also were displayed for each scale. Ratings were provided by moving a bar on a continuous slider scale. After providing and confirming ratings on both scales, the subsequent picture was presented.

#### EEG recording and analysis

EEG was recorded in a sound and light-attenuated laboratory using a 64-channel NeuroScan Ag/AgCl QuikCap connected to a SynAmps2 amplifier. Electrodes were placed according to the 10/20 international placement system, with an additional four electrodes for EOG monitoring. Impedances were kept below 5 kΩ. The signal was digitized at 1,000 Hz with 0.1-100 Hz acquisition mode in Neuroscan SCAN 4.4 software.

Continuous EEG recording was processed with EEGlab (Delorme & Makeig, 2004) and ERPlab (Lopez-Calderon & Luck, 2014). Electrodes were re-referenced offline to linked mastoids, and the signal was resampled to 256 Hz and low-pass filtered at 30 Hz. Movement artifacts were manually removed from the recording before the data was subjected to independent component analysis. After the removal of the eye-blink components, the signal was epoched. Three types of epochs were extracted and corrected for prestimulus baseline: target-locked 4,400-ms epochs (400-ms baseline), probe-locked 1,200-ms epochs (200-ms baseline), and picture-locked 1,200-ms epochs (200-ms baseline). Target and probe trials were averaged only from trials with correct answers. All of the trials were subjected to further ±100 μV artifact removal. The number of remaining trials can be found in Appendix B.

To examine the emotional response to the presented NAPS picture, Late Positive Potential was extracted as mean amplitude between 450 and 1,000 ms after the onset of the picture. To analyze the impact of emotional distraction on working memory, ERPs related to encoding (target P3: peak amplitude between 220 and 450 ms after target presentation), maintenance (NSW: mean amplitude between 1,000 and 4,000 ms), and retrieval (probe P3: early: peak amplitude between 230 and 430 ms; late: 430-750 ms after the probe onset) were extracted for each condition. A previous study that used the same paradigm reported that both target and probe P3 amplitudes, as well as emotional effects, were maximal over midline parietal electrodes (Li et al., 2010), thus P3 values were evaluated at Pz. The same study found broadly distributed NSW with emotional effects observed primarily on the midline electrodes, thus five (Fpz, Fz, Cz, Pz, Oz) midline electrodes were chosen for the analysis of late ERPs (LPP, NSW).

#### Statistical analysis

Ratings of NAPS pictures were analyzed using mixed factor repeated measures ANOVA with emotion (NEU vs. NEG) as a within subject factor and group (HC vs. SCZ) as a between-subject factor.

ANOVA with emotion (NEU vs. NEG) and site (Fpz, Fz, Cz, Pz, Oz) as within subject factors and group (HC vs. SCZ) as a between-subject factor was performed for LPP. ANOVA with emotion (NEU vs. NEG), task (VERBAL vs. SPATIAL), and site (Fpz, Fz, Cz, Pz, Oz) as a within subject factors and group (HC vs. SCZ) as a between-subject factor was performed for NSW.

Accuracy in the DMST task as well as target P3, NSW, and probe P3 values were analyzed using repeated measure ANOVA with emotion (NEU vs. NEG) and task (VERBAL vs. SPATIAL) as within subject factors and group (HC vs. SCZ) as a between-subject factor. The results were Greenhouse-Geisser corrected.

## Results

Participant’s sociodemographic and clinical characteristics are summarized in Table 1. Groups were matched for age and gender; however, patients had fewer years of education (Table 1).

**Table 1:** Sociodemographic and clinical characteristics of the participants

	SCZ M [SD]	range	HC M [SD]	range	
Sex	19 M - 9 F	-	19 M - 9F	-	n.s.
Age (yrs)	34.1 [7.2]	23-45	31.5 [6.9]	21-44	t = 1.4 n.s.
Edu (yrs)	14.3 [2.3]	11-17	16.2 [1.4]	11-17	t = 3.7**
PANSS-5Positive	6.3 [3]	4-14	-		
PANSS-5Negative	15 [4]	8-24	-		
PANSS-5Disorganization	8 [3]	4-13	-		
PANSS-5 Excitation	6 [2]	4-11			
PANSS-5 Depression	5 [3]	3-12			
CPZ Equivalent	374 [250]	0-1000	-		
Treatment	monotherapy n = 21 (risperidone n = 1, olanzapine n = 8, amisulpride n = 2, clozapine n = 2, quetiapine = 4, aripiprazole n = 5); polytherapy n = 6 (aripiprazole + olanzapine n = 2, aripiprazole + clozapine n = 2, amisulpride + olanzapine n = 2); no medication n = 1				

**p < 0.01

### Behavioral results

#### Picture ratings

NEG pictures were rated as more arousing (NEG: 5.39 ± 1.86 vs. NEU: 3.00 ± 1.39; F(1,53) = 161.8; *p* < 0.001) and more negative (NEG: 2.9 ± 0.8 vs. NEU: 5.5 ± 0.6 F(1,53) = 428.9; *p* < 0.001) than NEU pictures by all participants.

Patients rated all pictures (NEG and NEU) as more arousing (HC: 3.45 ± 1.44 vs. SCZ: 4.91 ± 1.16 F(1,53) = 17.3; *p* < 0.001) than controls. For valence ratings, the effect of group (HC: 4.3 ± 0.4 vs. SCZ: 4.0 ± 0.5 F(1,53) = 5.5; *p* < 0.05) was further modulated by emotion (F(1,53) = 5.9; *p* < 0.05) with similar ratings of valence for NEU pictures (HC: 5.4 ± 0.5 vs. SCZ: 5.5 ± 0.7; n.s.) and lower ratings of pictures for NEG pictures in patients (HC: 3.2 ± 0.7 vs. SCZ: 2.6 ± 0.7; *p* < 0.01) compared with controls.

#### DMST results

A significant effect of task was found (F(1,54) = 53.0; *p* < 0.001) with lower accuracy for the SPATIAL condition (VERBAL: 91% ± 6% vs. SPATIAL: 84% ± 10%). Patients had lower overall accuracy than controls (HC: 91% ± 5% SCZ: 84 ± 7% 50.3 F(1,54) = 18.7; *p* < 0.001). Additionally, an interaction between group and task was found, with between-group discrepancies being of larger magnitude for SPATIAL than for VERBAL (F(1.54) = 7.9; *p* < 0.01). A significant emotion by task interaction also was found, with significantly lower accuracy for NEG than for NEU only in the VERBAL condition (VERBAL: NEU: 92% ± 6% NEG: 90% ± 7%; SPATIAL: NEU: 84% ± 11% NEG: 84% ± 11% F(1,54) = 4.3; *p* < 0.05). Accuracy values for each condition in each group are shown in Table 2.

**Table 2:** Basic descriptive statistics of response accuracy (ACC) in both groups

Type of distraction	Type of instruction	SCZ	HC
		ACC [%]	ACC [%]
Neutral	Verbal	91+/-6	94+/-4%
	Spatial	78+/-10	89+/-8%
Negative	Verbal	88+/-8	93+/-4%
	Spatial	80+/-11	88+/-10%

### ERP results

**Fig. 1:**
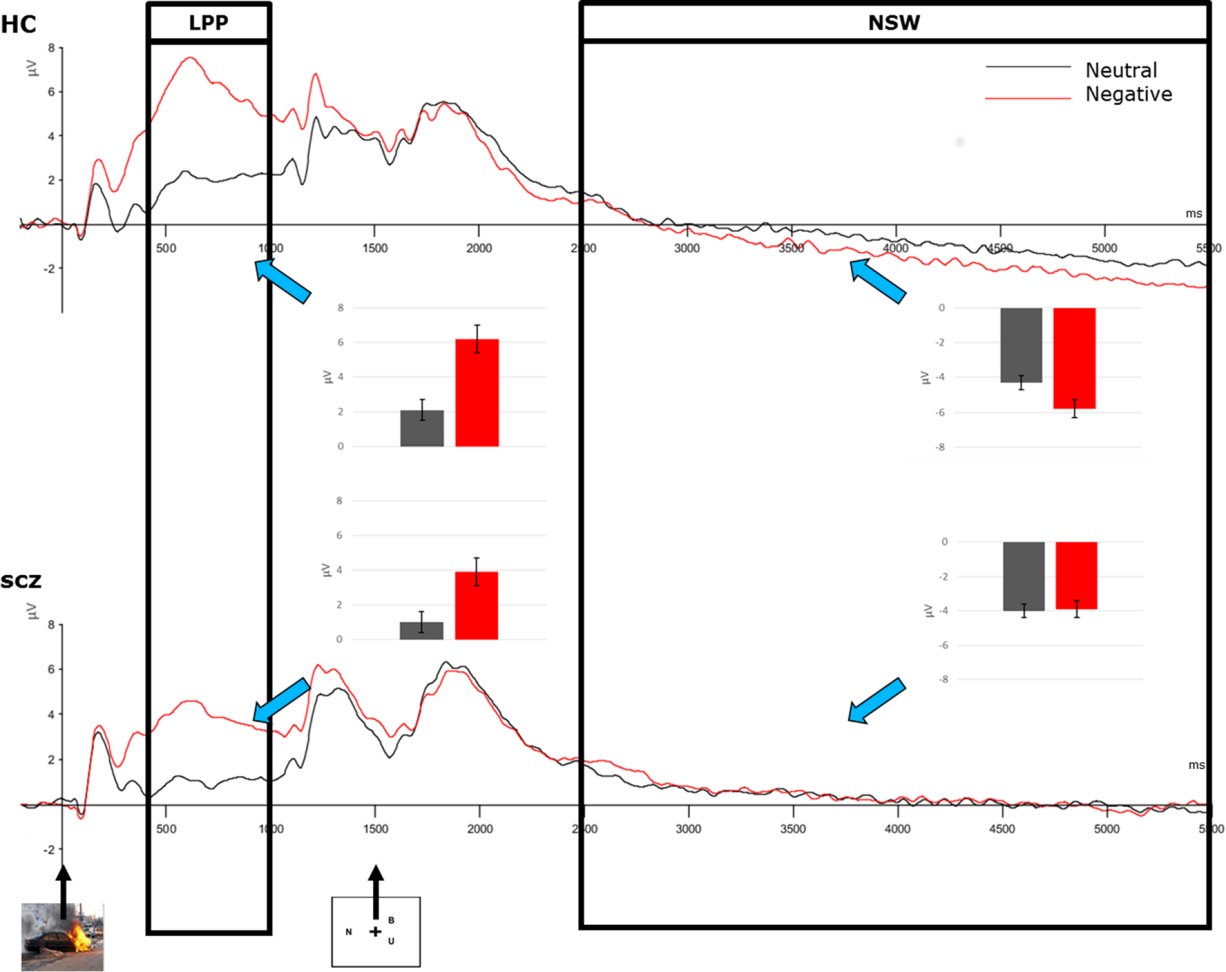
ERP waveform with LPP and NSW values for both conditions in both groups. Error bars are for mean values at the Pz electrode

Figure 1 shows the waveforms for distractor presentation, encoding, and maintenance stages of the DMST task.

#### Picture presentation

A main effect of emotion (F(1,54) = 93.5; *p* < 0.001) was observed, with larger mean LPP amplitudes for NEG than NEU stimuli (NEG: 3.3 ± 3.2 μV vs. NEU: 0.8 ± 2.3 μV). Furthermore, this was modulated by electrode placement (F(4,54) = 38.0; *p* < 0.001), with the strongest effects of emotion observed at Cz (mean difference between NEG and NEU: 3.6 ± 2.7 μV) and Pz (3.5 ± 2.4 μV). Differences between LPP to NEG and NEU gradually decreased toward anterior and posterior sites, with a minimal effect of emotion observed at Oz (0.8 ± 1.3 μV) and Fpz (1.9 ± 2.4 μV).

A main effect of site also was observed (F(4,54) = 56.6; *p* < 0.001), which was further modulated by group (F(4,54) = 5.1; *p* < 0.01): in HC, LPP amplitude was largest at Pz (4.1 ± 3.2 μV), whereas in SCZ, it was maximal at Cz (4.1 ± 4.4 μV).

No two-way (emotion by group F(1,54) = 1.6; n.s.) or three-way (emotion by site by group: F(4,54) = 2.3 n.s.) interactions were observed, thus suggesting similar processing of affective stimuli in both groups.

To further ensure that groups did not differ in terms of neurophysiological reaction to the affective stimuli, we used Bayesian statistics, which allow to infer directly which of the hypotheses better explains the observed data. Bayesian factors were calculated with JASP (https://jasp-stats.org/) for emotion x group (BF_01_ = 2.2) and emotion x group x site (BF_01_ = 21.5). In both cases, the null hypothesis explained the data better than the interaction hypothesis, which is reflected by a Bayesian factor greater than 1 for null versus interaction hypothesis.

#### Encoding

**Fig. 2:**
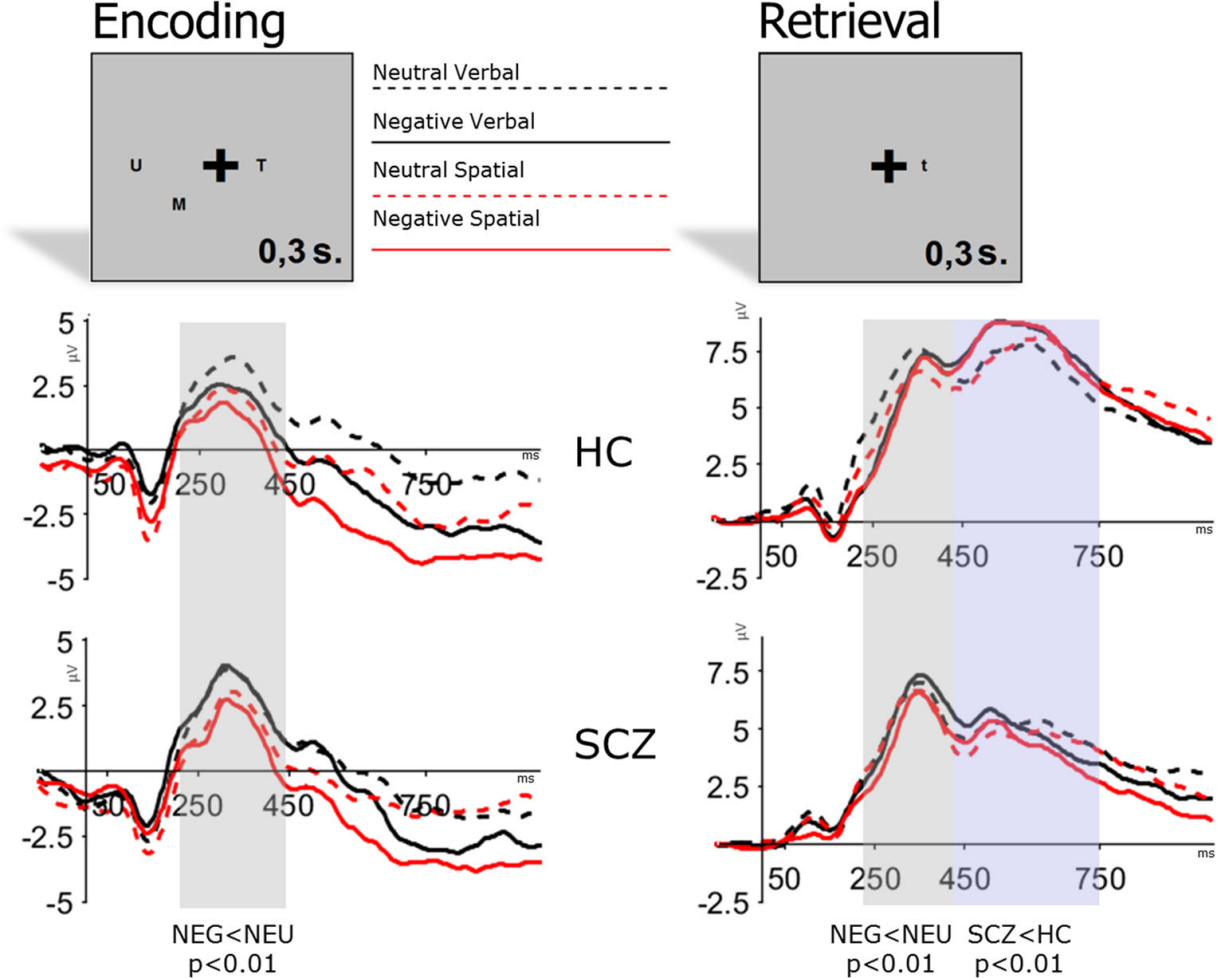
Impact of negative and neutral distraction on verbal and spatial working memory during the encoding and retrieval phases. Highlighted areas are for early and late P3

No significant emotion by group interaction was observed during the encoding phase. A main effect of emotion (F(1,54) = 11.1; *p* < 0.01) was observed, with higher target P3 peak amplitude for NEU than for NEG (NEU: 5.6 ± 3.6 μV vs. NEG: 4.7 ± 3.5 μV). No effects of the task or interaction between the task and other factors were observed during encoding. Waveforms for each condition during encoding are shown in the left panel of Fig. 2.

#### Maintenance

An interaction between emotion and group was observed (F(4,54) = 5.7; *p* < 0.05) during the maintenance phase: the effect of distraction type (NEU vs. NEG) was observed in HC (F(1,27) = 31.9; *p* < 0.001) but not in SCZ (F(1,27) = 0.9; n.s.). Main effects of emotion (F(1,54) = 15.6; *p* < 0.001) and site (F(4,54) = 153.4; *p* < 0.001) were observed during the maintenance, with more negative slow wave (NSW) for NEG than for NEU (NEU: −3.4 ± 1.7 vs. NEG: −4.4 ± 2.0) and maximally negative amplitudes at Cz (−6.4 ± 2.5 μV). Furthermore, an interaction between emotion and site was observed (F(4,54) = 28.4; *p* < 0.001), with more negative amplitudes for NEG than NEU pictures at all electrodes but Oz, where NEG was more positive than NEU pictures. No effect of the task or interaction between the task and other factors was observed during the maintenance phase.

Because significant discrepancies were observed between HC and SCZ in both behavioral ratings of distraction pictures and in overall DMST accuracy, the results were reexamined after inclusion of ratings and percent of DMST correct responses as covariates. ANCOVA results confirmed that a significant emotion by group interaction (F(1,48) = 5.5; *p* < 0.05) can be observed after controlling for both emotional and cognitive performance.

#### Retrieval

No significant emotion by group interaction was observed for early or late P3 during the retrieval phase. A main effect of emotion (F(1,54) = 10.3; *p* < 0.01) was observed for early P3 during retrieval. The early P3 peak had a higher amplitude for NEU than for NEG pictures (NEU: 9.3 ± 0.5 μV vs. NEG: 8.6 ± 0.5 μV). For the late P3, a main effect of group was observed (F(1,54) = 8.3; *p* < 0.01) with overall higher late P3 component amplitudes in HC than in SCZ. No effects of the task or interaction between the task and other factors were observed during the retrieval for any of the components. Waveforms for each condition during retrieval are shown in the right panel in Fig. 2.

Because a significant group effect was observed, similarly as in the case of NSW we reanalyzed the pattern of results for the late P3 values after inclusion of behavioral results and ratings of affective pictures. ANCOVA revealed no between-group differences in late P3 values after inclusion of behavioral parameters (F(1,48) = 1.8 n.s.).

## Discussion

This study examined the behavioral and neurophysiological markers of the influence of negative and neutral distracting stimuli on working memory performance in HC and SCZ. Analysis of the performance revealed that affective pictures had a negative impact on verbal working memory. At the same time, regardless of the type of task (VERBAL or SPATIAL) or distraction, SCZ presented lower accuracy in the DMST than HC. At the neurophysiological level, both groups showed similar neural activity while various emotional stimuli were presented. However, only healthy controls displayed enhanced neural activation (increased amplitude of NSW) related to working memory maintenance after the presentation of a highly salient negative distraction. This result suggests that healthy individuals, unlike patients with schizophrenia, recruit more resources to maintain information while faced with more salient distraction stimuli. At the same time, similar effects of emotional distraction were observed in both groups during working memory encoding and retrieval phases. Decreased neural activity also was found in SCZ during retrieval.

Interpretation of these results may be twofold. First, working memory deficits observed in SCZ (Reichenberg & Harvey, 2007) have been previously linked to inefficient task-related frontoparietal activation. Thus, there is a possibility that lack of NSW modulation in patients may be just a mere reflection of “hypofrontality” (decreased prefrontal activation during executive and working memory tasks in patients; Minzenberg, Laird, Thelen, Carter, & Glahn, 2009), especially given the overall lower DMST accuracy in SCZ. This view may be further supported by a previous observation that both frontal and parietal structures contribute to the NSW observed at the posterior scalp sites (Morgan et al., 2010). However, the DMST used in this study was modified to include emotional distraction pictures. Hence, the role of the frontoparietal network during the task is not only to support working memory processes but also to suppress distractor-related limbic activity. The heightened amplitude of NSW during NEG thus may be linked to increased neural activity associated with defying the impact of external salient distraction during the rehearsal of the target material. It also is important to notice that, unlike between-group differences for the retrieval-related late P3, the pattern of differences in NSW modulation was still observed after inclusion of the behavioral responses of participants as a covariate. Thus, we believe that working memory related “hypofrontality” is reflected in the late P3 component during retrieval, whereas the lack of NSW modulation during the maintenance stage is associated with failure to control emotional distractors in patients with schizophrenia. Our results support the view that the abnormal patterns of neural activity observed in neuroimaging studies on emotional interference in working memory in SCZ (Anticevic et al., 2011; Diaz et al., 2011) are driven by the failure of SCZ to deploy appropriate cognitive resources needed to inhibit affective distraction during the working memory maintenance phase.

It is important to notice that working memory-maintenance is the only phase of working memory in which no external stimuli is presented, and the sole role of the participant is to focus fully on the internal representation of the presented material. Thus, deficient inhibitory control in SCZ may become evident during working memory-maintenance when the suppression of any extraneous activity and implementation of an effective maintenance strategy becomes critical for internal rehearsal of the material.

Deficient allocation of cognitive resources during complex cognitive-emotional processing may be another mechanism that contributes to deficient control over affective distractors in schizophrenia. The fact that patients exhibited identical NSW as HC for NEU pictures but failed to enhance it for NEG pictures may be associated with SCZ displaying maximal neural activation when facing easy to intermediate levels task difficulty (NEU distraction). This interpretation of abnormal cognitive regulation in a complex cognitive-emotional task is supported by neurophysiological studies in which SCZ overly activated the neural mechanism when faced with tasks that have minimal cognitive demands (Gaspar et al., 2011; Missonnier et al., 2012) or resting state hyperactivity under no cognitive demands at all (Whitfield-Gabrieli et al., 2009). Neuroimaging studies also have revealed that SCZ may activate a widespread network of brain structures as a compensatory mechanisms, with the most significant over activations in the dorsolateral prefrontal cortex (dlPFC), to achieve similar results as HC in less demanding working memory tasks (Potkin et al., 2009). These findings suggest that SCZ may have difficulty with upregulating the working memory system due to central executive deficits.

However, some limitations of the current study should have be pointed out. First, we did not assess the cognitive profile of our participants. This would have allowed controlling for working memory levels in SCZ and in HC. Yet, participants from both groups had very high accuracy in the task, even though healthy participants outscored the patients. We also attempted to control for cognitive difficulties in SCZ by using behavioral performance as a covariate. Second, 27 of 28 of the patients in the sample were using antipsychotic medication at the time of the study. While both executive control deficits (Fatouros-Bergman, Cervenka, Flyckt, Edman, & Farde, 2014) and reduced frontoparietal activity (Andreasen et al., 1997) have been found in drug naïve patients with schizophrenia, the impact of neuroleptic medication on the current results cannot be fully ruled out. Another issue that should be considered is the fact that SCZ rated the distractor stimuli as more arousing than HC, thus suggesting that both groups may have been differentially affected by the neutral and negative pictures. Nonetheless, we observed similar LPP enhancement in response to negative stimuli in both groups of participants. Late Positive Potential (LPP) is widely regarded as an ERP indicator of the amount of sustained attention to affective visual stimuli (Liu et al., 2012; Olofsson, Nordin, Sequeira, & Polich, 2008). Intact LPP modulation was found in a number of studies that presented emotional stimuli to SCZ during various task designs (Horan, Foti, Hajcak, Wynn, & Green, 2012; Horan, Wynn, Kring, Simons, & Green, 2010; Pinheiro et al., 2013). Furthermore, in our previous study, we found a similar pattern of behavioral and neural response to the subset of the stimuli used in this study, which was presented during an oddball task (Okruszek et al., 2016). Thus, despite differences in the behavioral ratings of the stimuli, we believe that the fact that both group showed similar LPP modulation reflects intact emotional reactivity in SCZ and is proof of similar reactions to affective stimuli in SCZ and HC. This notion is reinforced by the fact that neurophysiological results remained unchanged after inclusion of the behavioral ratings as a covariate. Furthermore, despite having previously shown that patients and controls display similar behavioral and neurophysiological response to the social and nonsocial affective stimuli (Okruszek et al., 2016) that were used in the current study, it cannot be ruled out that the presence of social content still could have impacted our current results. Another possible limitation is the fact that, to limit the length of the procedure, only neutral stimuli were used as a contrast for negative distraction, while it has been shown that positive and negative distraction may have a different effect on working memory in HC, which can be linked to dissociations in brain activations (Iordan & Dolcos, 2015). Moreover, studies have shown that SCZ may present abnormalities in responding to emotional stimuli of specific valence (Horan et al., 2010); thus, further studies on the effect of emotional interference in working memory should examine not only arousal but also valence effects. Additionally, further studies should use simultaneous EEG-fMRI methodology to elucidate if the NSW abnormalities observed in this study can be directly linked to the abnormal frontoparietal activations previously observed during the retention phase of cognition-emotion paradigms in SCZ.

In conclusion, the results of this study suggest that cognitive deficits in people with schizophrenia can be amplified by impaired control of emotional information processing. In addition to replicating the “hypofrontality” effects (reduced late P3 at retrieval), which may be linked to lower working memory capacity in SCZ, we also have observed decreased interference control during working memory-maintenance (lack of NSW modulation) in patients. Thus, strategies that could help patients to strengthen cognitive regulation during working memory-maintenance and therapies to decrease the impact of strong emotion should be proposed and evaluated. Studies looking at manipulating this effect may want to target the individual’s awareness of this effect and develop a psychoeducational intervention considering the effect of emotion on cognition.

## Electronic supplementary material

Figure S1. Timeline for the presentation of stimuli during the DMST task. (PNG 110 kb)

ESM 1 (DOCX 13 kb)
