# Supplementary figures and images for: Using ERPs to explore the impact of affective distraction on working memory stages in schizophrenia
Source: Cogn Affect Behav Neurosci. 2018 Apr 13;18(3):437–46. doi: 10.3758/s13415-018-0578-4 (PMC5962617; doi:10.3758/s13415-018-0578-4)

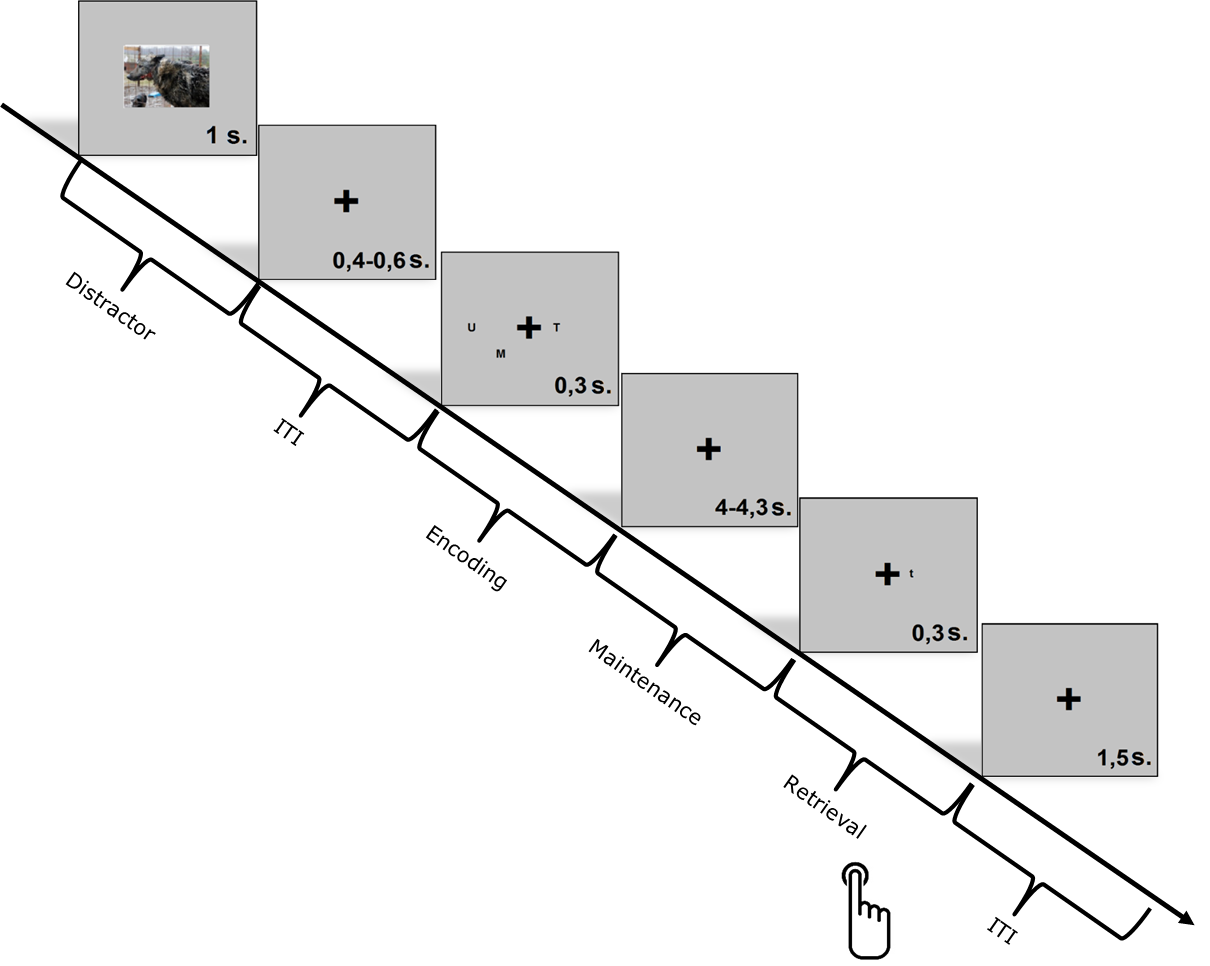

Supplement: Supplementary file 1 — Timeline for the presentation of stimuli during the DMST task. (PNG 110 kb) [file 13415_2018_578_MOESM1_ESM.png]
