# Supplementary material for: Using ERPs to explore the impact of affective distraction on working memory stages in schizophrenia
Source: Cogn Affect Behav Neurosci. 2018 Apr 13;18(3):437–46. doi: 10.3758/s13415-018-0578-4 (PMC5962617; doi:10.3758/s13415-018-0578-4)
Supplement: Supplementary file 2 — (DOCX 13 kb) [file 13415_2018_578_MOESM2_ESM.docx]

Supplementary Table 1. List of NAPS pictures used in the study

| Neutral | | Negative | |
| --- | --- | --- | --- |
| Animals 088 | Faces 060 | Animals 001 | Faces 003 |
| Animals 104 | Faces 078 | Animals 033 | Faces 008 |
| Animals 105 | Faces 182 | Animals 037 | Faces 009 |
| Animals 119 | Faces 184 | Animals 039 | Faces 010 |
| Animals 146 | Faces 186 | Animals 048 | Faces 028 |
| Animals 169 | Faces 190 | Animals 056 | Faces 034 |
| Objects 177 | Faces 192 | Animals 074 | Faces 144 |
| Objects 187 | Faces 194 | Animals 075 | Faces 150 |
| Objects 189 | Faces 196 | Animals 077 | Faces 157 |
| Objects 196 | Faces 203 | Animals 078 | Faces 158 |
| Objects 209 | Faces 205 | Objects 001 | Faces 272 |
| Objects 210 | Faces 209 | Objects 003 | Faces 280 |
| Objects 212 | Faces 220 | Objects 006 | Faces 283 |
| Objects 222 | Faces 224 | Objects 013 | Faces 284 |
| Objects 223 | Faces 305 | Objects 022 | Faces 287 |
| Objects 224 | Faces 311 | Objects 109 | Faces 366 |
| Objects 234 | Faces 315 | Objects 120 | Faces 368 |
| Objects 237 | Faces 326 | Objects 125 | Faces 370 |
| Objects 238 | Faces 331 | Objects 126 | People 084 |
| Objects 244 | People 097 | Objects 127 | People 124 |
| Objects 245 | People 122 | Objects 132 | People 127 |
| Objects 246 | People 146 | Objects 139 | People 145 |
| Objects 247 | People 149 | Objects 143 | People 198 |
| Objects 248 | People 150 | Objects 148 | People 215 |
| Objects 252 | People 151 | Objects 148 | People 220 |
| Objects 254 | People 153 | Objects 149 | People 222 |
| Objects 279 | People 158 | Objects 154 | People 227 |
| Objects 292 | People 159 | Objects 158 | People 239 |
| Objects 308 | People 162 | Objects 283 | People 240 |
| Objects 311 | People 166 | Objects 328 | People 246 |

Supplementary Table 2. Number of trials that were averaged for each condition.

|  | HC NEU  M [SD] | HC NEG  M [SD] | SCZ NEU  M [SD] | SCZ NEG  M [SD] |
| --- | --- | --- | --- | --- |
| Affective Pictures | 100.9 [16.5] | 100.9 [17.8] | 95.9 [20.8] | 95.8 [20.4] |
| Verbal Encoding | 51.8 [6.9] | 48.7 [8.1] | 48.5 [10.0] | 45.8 [9.9] |
| Spatial Encoding | 47.6 [7.7] | 48.7 [8.1] | 43.1 [9.0] | 41.1 [10.9] |
| Verbal Retrieval | 54.9 [4.0] | 53.8 [3.3] | 52.9 [4.2] | 51.3 [4.8] |
| Spatial Retrieval | 52.0 [5.2] | 51.8 [5.8] | 45.3 [6.6] | 46.3 [7.1] |
